# Effectiveness of immune therapy combined with chemotherapy on the immune function and recurrence rate of cervical cancer

**DOI:** 10.3892/etm.2015.2217

**Published:** 2015-01-26

**Authors:** BIN CHEN, LIFEN LIU, HAIYAN XU, YIJIN YANG, LING ZHANG, FENGCHUN ZHANG

**Affiliations:** 1 Department of Oncology, Suzhou Kowloon Hospital, Shanghai Jiao Tong University School of Medicine, Suzhou, Jiangsu 215000, P.R. China; 2 Department of Gynecology and Obstetrics, Second Affiliated Hospital of Soochow University, Suzhou, Jiangsu 215004, P.R. China

**Keywords:** dendritic cell-cytokine-induced killer cell, cisplatin, cervical cancer, immune function, recurrence rate

## Abstract

The aim of this study was to compare the immune function of patients with cervical cancer and the cancer recurrence rate in patients treated with biological immune therapy combined with chemotherapy or with chemotherapy only. A total of 79 postoperative patients with cervical cancer participated in the present study. They were randomly divided into a control group and an experimental group. Patients in the control group were treated with cisplatin chemotherapy. Patients in the experimental group were treated with dendritic cell-cytokine-induced killer (DC-CIK) cells combined with cisplatin chemotherapy. The CD3^+^, CD4^+^, CD8^+^, CD16^+^, CD56^+^ and CD4^+^CD25^+^ cell ratios in peripheral blood, and the expression levels of perforin, granzyme B (GraB) and CD107a of peripheral blood mononuclear cells (PBMCs) in all patients prior to and following treatment were observed. The changes of immune function and recurrence rate between these two groups prior to and following treatment were compared. Prior to treatment, the lymphocyte ratio had no significant difference between the two groups (P>0.05). Following treatment, the lymphocyte ratio in the experimental group was significantly higher than that in the control group (P<0.05). The positive expression levels of perforin, GraB and CD107a of PBMCs in the experimental group following treatment were significantly higher than those prior to treatment and those of the control group (P<0.05). The cumulative recurrence rate in the experimental group was significantly lower than that in the control group (P<0.05). In conclusion, in postoperative patients with cervical cancer, treatment with DC-CIK cells combined with cisplatin chemotherapy significantly improved the immune function, reduced the recurrence rate and prolonged the survival time of the patients.

## Introduction

Cervical cancer is one of the most common gynecologic malignant tumors. It mainly results from chronic infection with human papillomavirus (HPV), which leads to abnormal metaplasia and subsequent cancer. In recent years, the age of incidence of cervical cancer has been decreasing (1,2). Currently, the clinical treatment of cervical cancer is focused on surgery and chemotherapy, sometimes in combination with traditional Chinese medicine treatment. However, the cure rate in patients with advanced cervical cancer remains very low and the recurrence rate is high (3). Cancer patients often have severe cellular immune function defects, and cervical cancer patients are no exception. Therefore, improving the cellular immunity functions of cancer patients is essential to improve their survival rate and cure rate. In recent years, the application of biological treatment technology in tumor immunotherapy has been constantly expanding and gradually accepted by medical personnel and patients. This technology has become the fourth main treatment option for tumors, following surgery, radiotherapy and chemotherapy. Cytokine-induced killer (CIK) cells have become the preferred type of cells used in antitumor adoptive cell immunotherapy. CIK cells are heterogeneous cells that can be generated from human peripheral blood mononuclear cells (PBMCs) by culturing in the presence of a variety of cytokines *in vitro* (4). CIK cells have the advantages having the strong antitumor activity of T lymphocytes and a non-major histocompatibility complex (MHC)-restricted tumoricidal effect (5–7). Dendritic cells (DCs) are currently the most powerful antigen-presenting cells (APCs) that have been discovered. They can induce antigen-specific immune responses. A co-culture of DCs and CIK cells has the advantages of fast proliferation, high cytotoxicity and a broad tumor-killing spectrum (8). In recent years, clinical studies concerning the use of biological immunotherapy in the treatment of cancer have been increasing; however, few of them have focused on cervical cancer (9). Through comparing the efficacy of chemotherapy alone with that of biological immune therapy combined with chemotherapy on patients with cervical cancer, the current study explored the effectiveness of immunotherapy combined with chemotherapy on the immune function of patients with cervical cancer and the recurrence rate of the cancer.

## Subjects and methods

### Clinical data

A total of 79 patients with cervical cancer in Suzhou Kowloon Hospital (Suzhou, China) from March 2008 to March 2010 participated in the study. The selection criteria of the patients were as follows: i) diagnosed as cervical cancer by pathology; ii) between the age of 30 and 70 years; iii) without organ lesions, e.g., of the liver, kidney or heart; iv) without cardiovascular and psychiatric disorders; and v) signed an acknowledgment agreement. The patients were randomly divided into a control group and an experimental group. The patients of the control group were treated with chemotherapy, while the patients of the experimental group were treated with biological immune therapy combined with chemotherapy. The control group consisted of 39 cases (age, 51.9±16.8 years), 30 of whom had squamous cell carcinoma and nine of whom had adenocarcinoma. According to International Federation of Gynecology and Obstetrics (FIGO) stage classification, there were 16 cases in Stage IIa, 11 cases in Stage IIb, seven cases in Stage IIIa, four cases in Stage IIIb, and one case in Stage IV. The experimental group consisted of 40 cases (age, 52.4±17.1 years), 31 of whom had squamous cell carcinoma and nine of whom had adenocarcinoma. According to FIGO stage classification, there were 15 cases in Stage IIa, 12 cases in Stage IIb, eight cases in Stage IIIa, four cases in Stage IIIb and one case in Stage IV. There were no significant differences between the two groups in age, clinical stage or the degree of tumor invasion (P>0.05). This study was conducted in accordance with the Declaration of Helsinki and with approval from the Ethics Committee of Suzhou Kowloon Hospital, Shanghai Jiao Tong University School of Medicine. Written informed consent was obtained from all participants.

### Preparation of DC-CIK cells

Prior to chemotherapy, 1.5–4.0×10^9^ PBMCs were collected from each patient. During the collection of the PBMCs, 60 ml plasma was isolated to prepare for cell reinfusion. The collected PBMCs were centrifuged at 2,500 × g for 10 min, and the supernatant was decanted. The sediment was washed with phosphate-buffered saline (PBS) twice and then resuspended. After that, the PBMCs were mixed with lymphocyte separation medium (Dingguo Biotechnology Co., Beijing, China) in a 1:1 ratio, centrifuged at 3,000 × g for 15 min and the supernatant was decanted. The PBMCs were washed with normal saline three times, and then resuspended in serum-free medium to modulate the cells. The suspension was divided into two parts in the ratio of 1:9. The cell density of the small part of the suspension was adjusted to 1–2×10^7^/ml. Then, the suspension was seeded in 6-well plates (2 ml/plate), adherently cultured for 2 h in an incubator and then the non-adherent cells were sucked out. Serum-free medium containing granulocyte-macrophage colony-stimulating factor (GMCSF; 800 U/ml; Dingguo Biotechnology Co.) and interleukin (IL)-4 (500 U/ml; Dingguo Biotechnology Co.) was added for culturing DCs, and replaced every 3 days. On day 7, tumor necrosis factor (TNF)-α (1,000 U/ml; Dingguo Biotechnology Co.) was added to promote maturation. The large part of the suspension was mixed with interferon (INF)-γ (1,000 U/ml; Dingguo Biotechnology Co.) and used as CIK serum-free medium, in which IL-1α (100 U/ml), IL-2 (500 U/ml) and CD3 monoclonal mouse anti-human antibodies (Boster Biotechnology Co., Wuhan, China) were added from day 2, and replaced every 3 days. On day 8, the mature DCs were collected and divided into two portions. One portion of the DCs was washed with normal saline 3 times and then resuspended in 2 ml autologous plasma. This was then used for administration to patients by superficial lymph node or subcutaneous injection. The other portion of the DCs was mixed and co-cultured with CIK cells at a ratio of 1:10 for 7 days to construct the DC-CIK cell mixture. Starting from day 8, DC-CIK cells were reinfused to patients once a day for a total of four times and the total number of cells was 2–3.5×10^10^. Prior to reinfusion, all cells underwent bacterial, fungal and endotoxin detection tests and were found to be negative.

### Therapeutic methods

Patients in the control group were given conventional chemotherapy with cisplatin (20 mg per day; Haoshen Pharmaceutical Co., Nanjing, China). This involved dissolving 20 mg cisplatin in 250 ml normal saline, and then administered by intravenous drip within 2 h with the avoidance of light. One course of treatment lasted for 10 days. Patients in the experimental group underwent the collection of PMBCs 1 day prior to chemotherapy, which were used for co-culture to generate DC-CIK cells. From the second day, the experimental patients were given the same chemotherapy as that given to the patients in the control group. However, the experimental patients were also given a reinfusion of DC-CIK cells after the chemotherapy. Three months later, all patients from these two groups were given a second course of treatment.

### Observation indices and judgment of curative effect

Prior to treatment and 2 weeks after treatment, 4 ml peripheral blood was collected from each patient of the two groups. The CD3^+^, CD4^+^, CD8^+^, CD16^+^, CD56^+^ and CD4^+^CD25^+^ cell ratios in peripheral blood were detected by flow cytometry and the expression levels of perforin, granzyme B (GraB) and CD107a in the PBMCs were observed. The cumulative relapse rate, the cumulative survival rate and the median survival time of the patients were also analyzed.

### Statistical analysis

Statistical analysis was performed using SPSS software, version 13.0 (SPSS Inc., Chicago, IL, USA). Continuous variables are presented as the mean ± standard deviation and were tested with the Student’s t-test. Categorical variables were tested with the Chi-square test. Cumulative recurrence rate and cumulative survival rate were compared with the Kaplan-Meier method and log-rank test. P<0.05 was considered to indicate a statistically significant difference.

## Results

### Changes in peripheral blood T-lymphocyte subsets in the two groups prior to and following treatment

**Figure 1:**
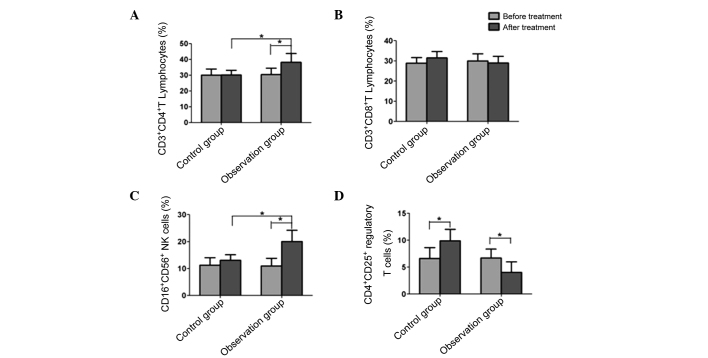
Comparison of lymphocyte subsets in peripheral blood between the control and experimental groups. (A) CD3^+^CD4^+^ and (B) CD3^+^CD8^+^ T lymphocytes, (C) CD16^+^CD56^+^ natural killer cells and (D) CD4^+^CD25^+^ regulatory T cells. ^*^P<0.05 represents a significant difference.

Prior to treatment, the CD3^+^CD4^+^, CD3^+^CD8^+^, CD16^+^CD56^+^ and CD4^+^CD25^+^ cell levels exhibited no significant differences between the experimental and control groups (P>0.05). In the patients of the control group, the CD3^+^CD4^+^, CD3^+^CD8^+^ and CD16^+^CD56^+^ cell levels had no significant differences prior to and following treatment, but the CD4^+^CD25^+^ regulatory T-cell ratio significantly increased after treatment (P<0.05). Following treatment, the CD3^+^CD4^+^ and CD16^+^CD56^+^ cell levels in the experimental group were significantly higher than those prior to treatment (P<0.05), but the CD3^+^CD8^+^ cell level did not significantly differ from that prior to treatment. In addition, the CD4^+^CD25^+^ regulatory T-cell ratio following treatment was significantly reduced in the experimental group compared with that prior to treatment (Fig. 1).

### Changes in the expression levels of perforin, GraB and CD107a in the PBMCs of the two groups prior to and following treatment

**Figure 2:**
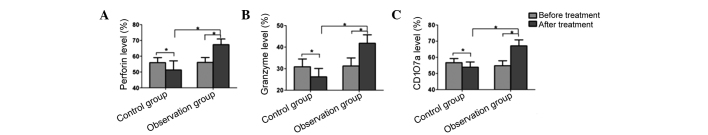
Comparison of the expression levels of (A) perforin, (B) granzyme B and (C) CD107a in peripheral blood mononuclear cells between the control and experimental groups. ^*^P<0.05 represents a significant difference.

The expression levels of perforin, GraB and CD107a in the PBMCs of the control group after treatment were significantly decreased compared with those prior to treatment (P<0.05). However, in the experimental group, the expression levels of perforin, GraB and CD107a significantly increased after treatment compared with those prior to treatment (P<0.05; Fig. 2).

### Comparison of the cumulative recurrence rate and cumulative survival rate between the two groups

**Figure 3:**
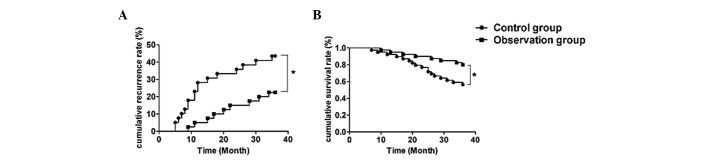
Comparison of the cumulative (A) recurrence rate and (B) survival rate between the control and experimental groups. ^*^P<0.05 represents a significant difference.

Patients from the two groups were followed up for more than 3 years after treatment. Eighteen recurrent cases were found in the control group but only nine recurrent cases were found in the experimental group. Kaplan-Meier curves demonstrated that the cumulative 1-year, 2-year and 3-year recurrence rates in the control group were 28.2% (11 cases), 35.9% (14 cases) and 46.2% (18 cases), respectively. In the experimental group, the cumulative recurrence rates were 5% (2 cases), 15% (6 cases) and 22.5% (9 cases), respectively. The cumulative recurrence rates between the control and experimental groups were significantly different when compared by log-rank test (P<0.05). The cumulative 1-year, 2-year and 3-year survival rates in the control group were 92.31, 76.92 and 56.41%, respectively. In the experimental group, the cumulative survival rates were 97.25, 90 and 80%, respectively. Log-rank test showed that the cumulative survival rate of the experimental group was significantly different from that of the control group (P<0.05; Fig. 3).

## Discussion

Cervical cancer is one of the most common malignant tumors of females. The incidence rate of this cancer is second only to that of breast cancer, but the mortality rate is the highest among the malignant gynecological tumors (10). In the treatment of cervical cancer, the high recurrence rate following treatment is a major problem. Currently, platinum chemotherapy-based comprehensive treatment is the preferred method for treating recurrent cervical cancer, but the total efficiency is only 20–30% (11). Moreover, since the tumor volume is relatively large in the advanced stages, patients are susceptible to immune suppression during the chemotherapy process (12,13). Therefore, a feasible and acceptable treatment strategy is urgently required for the clinical treatment of advanced cervical cancer. In recent years, numerous clinical data have shown that the immune function of patients with cervical cancer is suppressed significantly due to disorders of cellular immune function (14). This phenomenon is particularly common in patients with advanced cancer (15).

Bio-immunotherapy has been widely applied to treat different types of tumors. Its effectiveness in killing and inhibiting tumors and advantage in improving the immune function of patients undergoing chemotherapy have been demonstrated by clinical researchers (16–18). Yang *et al* (19) applied autologous CIK cell infusion combined with cisplatin therapy to treat 20 patients with advanced cervical cancer and observed the results after one month. The authors found that the TH1/TH2 immune index and the number of natural killer cells increased significantly following the combined treatment, compared with those following cisplatin chemotherapy alone. Their study demonstrated that combined treatment with CIK cells and chemotherapy was able to improve the immune function of patients with cervical cancer; however, follow-up results and the 3-year recurrence rate were lacking. Long-term follow-up of the effectiveness of CIK cell treatment combined with chemotherapy is necessary. Zhu *et al* (20) applied a combined treatment comprising autoimmune cells and radiotherapy to treat cervical cancer. The results demonstrated that the immune function of the patients that received the combination therapy was significantly higher than that of the patients treated with radiotherapy alone. In addition, 5-year follow-up results showed that the quality of life in the majority of the patients that received the combined treatment was significantly improved, and the Karnofsky score and the overall 1-year, 2-year and 5-year survival rates of the patients treated with the combination therapy were significantly higher than those of the patients treated with radiotherapy alone. A study conducted by Zhu *et al* (20) demonstrated that radiotherapy combined with immune cell therapy for the treatment of cervical carcinoma was able to enhance the immune function of patients and prolong their survival periods.

Laurin *et al* (21) observed that CIK cells expressed high levels of perforin and GraB, and so were able to induce tumor cell apoptosis through an extracellular apoptosis pathway. Hackstein and Thomson (22) revealed that the cytotoxic activity of CIK cells was remarkably enhanced following co-culture with DCs and stimulation by DCs. It was demonstrated that co-cultures of DCs and CIK cells not only have non-MHC-restricted CIK cell cytotoxicity but also are able to stimulate the MHC-restricted cytotoxic effect mediated by antigen-loaded DC cells. Therefore, the specific killing effects were enhanced. In the present study, the effectiveness of a DC-CIK cell co-culture combined with cisplatin chemotherapy in the treatment of cervical cancer was observed, and it was found that the CD3^+^CD4^+^ and CD16^+^CD56^+^ cell levels in patients following treatment were significantly higher than those prior to treatment, but the proportion of CD4^+^CD25^+^ regulatory T cells significantly decreased following treatment (P<0.05), which corresponds well with the results of Yang *et al* (23). Moreover, in the present study, it was observed that the perforin, GraB and CD107a positive expression rates were significantly higher in the combined treatment group than in the control group (P<0.05), and the cumulative 1-year, 2-year and 3-year recurrence rates were significantly reduced in the combined treatment group compared with those in the control group (P<0.05).

In conclusion, this study confirmed the effectiveness of biological immune treatment, particularly biological immune treatment combined with chemotherapy, in the treatment of tumors, and provides further evidence to support its clinical application in the treatment of cervical cancer.
